# Megavoltage Radiosensitization of Gold Nanoparticles on a Glioblastoma Cancer Cell Line Using a Clinical Platform

**DOI:** 10.3390/ijms21020429

**Published:** 2020-01-09

**Authors:** Farasat Kazmi, Katherine A. Vallis, Balamurugan A. Vellayappan, Aishwarya Bandla, Duan Yukun, Robert Carlisle

**Affiliations:** 1 Early Phase Clinical Trials Unit, Department of Oncology, University of Oxford, Oxford OX3 7LE, UK; 2 CRUK/MRC Oxford Institute for Radiation Oncology, University of Oxford, Oxford OX3 7LE, UK; katherine.vallis@oncology.ox.ac.uk; 3 Department of Radiation Oncology, National University Cancer Institute (NCIS), Singapore 119228, Singapore; bala_vellayappan@nuhs.edu.sg; 4 Singapore Institute for Neurotechnology (SINAPSE), National University of Singapore (NUS), Singapore 117456, Singapore; aishbandla22@gmail.com; 5 Department of Chemical and Biomolecular Engineering, National University of Singapore (NUS), Singapore 117456, Singapore; yukunduan@u.nus.edu; 6 Department of Biomedical Engineering, University of Oxford, Oxford OX3 7DQ, UK; robert.carlisle@eng.ox.ac.uk

**Keywords:** nanoparticles, glioblastoma multiform, radiosensitizers, external beam radiotherapy

## Abstract

Gold nanoparticles (GNPs) have demonstrated significant dose enhancement with kilovoltage (kV) X-rays; however, recent studies have shown inconsistent findings with megavoltage (MV) X-rays. We propose to evaluate the radiosensitization effect on U87 glioblastoma (GBM) cells in the presence of 42 nm GNPs and irradiated with a clinical 6 MV photon beam. Cytotoxicity and radiosensitization were measured using MTS and clonogenic cellular radiation sensitivity assays, respectively. The sensitization enhancement ratio was calculated for 2 Gy (SER_2Gy_) with GNP (100 μg/mL). Dark field and MTS assays revealed high co-localization and good biocompatibility of the GNPs with GBM cells. A significant sensitization enhancement of 1.45 (*p* = 0.001) was observed with GNP 100 μg/mL. Similarly, at 6 Gy, there was significant difference in the survival fraction between the GBM alone group (mean (M) = 0.26, standard deviation (SD) = 0.008) and the GBM plus GNP group (M = 0.07, SD = 0.05, *p* = 0.03). GNPs enabled radiosensitization in U87 GBM cells at 2 Gy when irradiated using a clinical platform. In addition to the potential clinical utility of GNPs, these studies demonstrate the effectiveness of a robust and easy to standardize an in-vitro model that can be employed for future studies involving metal nanoparticle plus irradiation.

## 1. Introduction

Glioblastoma (GBM) is one of the most common and aggressive brain cancers in adults, affecting 3 in 100,000 individuals worldwide annually [1]. The current standard of care utilizes a multidisciplinary approach which involves maximal safe tumor resection followed by adjuvant radiotherapy and temozolamide (TMZ), a radiosensitizer [2]. Despite standard treatment, GBM invariably recurs with median progression-free survival ranging from 5.5 to 13.0 months [2]. The majority of relapses occur within the high dose radiation treatment field [3]. However, the radiation dose delivered to the tumor site is limited by the tolerance of the surrounding healthy brain tissue and, hence, there is an urgent need for novel therapeutics to improve clinical outcomes in these patients. 

A major appeal of nanoparticles (NPs) is their ability to deliver cytotoxic agents to the tumor site with increased precision and effectiveness via a phenomenon known as the enhanced permeability and retention effect (EPR) [4]. Although the true scale of EPR is a topic of contention, it represents a substantial improvement over the delivery of free drugs, potentially allowing a higher percentage of the active agent to reach its target, thereby enabling reduced dosing and lower off-target effects [5]. In recent years, gold nanoparticles (GNPs) have also shown great promise to be used as novel radiosensitizers [6,7]. Extensive preclinical studies have demonstrated significant local enhancement of the absorbed dose by the inclusion of GNPs compared to kilovoltage (kV) X-rays alone [8,9,10]. Indeed, Hainfield was the first to demonstrate the radiosensitization effect of 1.9 nm GNPs with 250 kV X-rays using an in vivo mouse model. Mice irradiated together with GNPs had an 86% chance of 1-year survival contrasting with 20% for X-rays alone [9]. Similarly, Dorsey’s group also demonstrated a dose enhancement of 30% with 13 nm GNPs incubated in U251 GBM cells when targeted with a 150 kV X-ray [11]. These findings have been further validated with existing in vitro and in vivo experiments using different cancer cell lines [12,13]. The phenomenon driving the impact of GNP is known as the photoelectric effect and has been attributed to the high atomic number of gold (Z = 79), resulting in a high mass energy coefficient relative to soft tissue [9]. More specific to U87 GBM cells, Chen recently reported a dose enhancement of 1.37 with bovine serum albumin capped 28 nm GNPs incubated in U87 GBM when irradiated with a 160 kV X-ray [14]. Using an in vivo mouse model, he demonstrated tumor regression by approximately 35% compared to RT alone [14].

However, in a clinical setting, kilovoltage X-rays have limited utility in radiotherapy as they have low dose depth penetration and are unable to deposit a radiation dose to deep-seated tumor sites [6]. To overcome this, megavoltage (MV) X-rays, in the range of 4 to 10 MV, are used as they provide deeper dose penetration [7]. Predictive models, such as the Monte Carlo simulation, have shown that no significant radiosensitization effects occur with GNPs in the MV range [6,7]. However, recent in-vitro experiments have demonstrated varying dose enhancements with GNPs (between 1.16 and 1.5), questioning the validity of these predictive models [15,16,17]. To account for these differences, it has been proposed that there may be an underlying biological effect exerted by GNPs which has not been well characterized [10]. 

To date, there is little standardization of the RT techniques and models which have been used to produce MV X-rays to conduct these experiments; hence, comparison between studies is challenging [18]. A lack of clarity is a consequence of all these factors, and the clinical translation of GNPs as radiosensitizers has faced significant setbacks. The primary aim of this study is to investigate the radiosensitization effects of GNPs on U87 human glioblastoma (GBM) cells using a 6 MV X-ray generated via the use of an easy to establish and standardized clinical linear accelerator (LINAC). 

## 2. Results

### 2.1. Fabrication and Characterization of GOLD Nanoparticles

**Figure 1:**
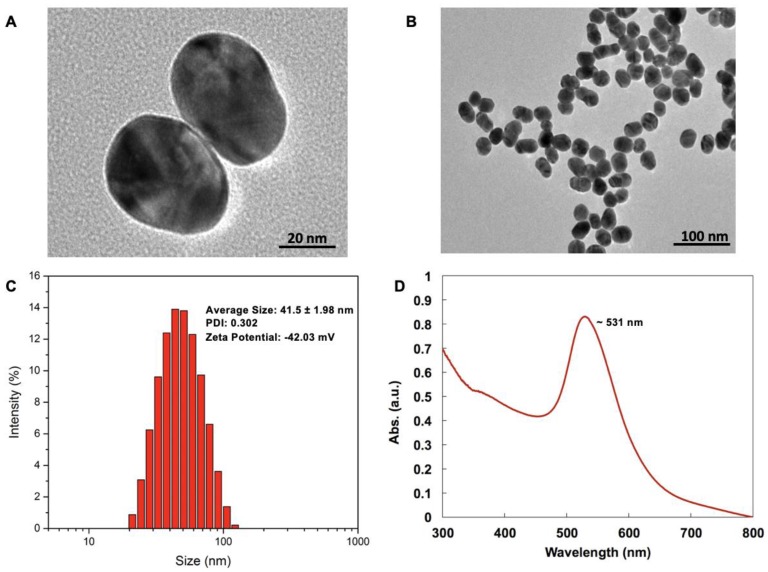
Gold nanoparticle characterization. Figure 1**A**,**B** shows images from the transmission electron microscopy of GNPs at 25,000× and 40,000× magnification, respectively, having a diameter of approximately 42 nm. Figure 1**C** represents the dynamic light scattering measurement of GNPs and shows that the sample was monodispersed. Figure 1**D** shows the UV–vis absorption spectrum of GNPs, with characteristic surface plasmon resonance at 531 nm.

Figure 1A,B shows TEM images of GNPs. In terms of size distribution, the polydispersity index (PDI) was 0.302 with a size of approximately 41.5 ± 1.98 nm, as shown by Figure 1C. A zeta potential of −42.03 mV further confirmed that GNPs in this sample were electrically stable, ensuring good colloidal dispersion. UV–vis spectroscopy showed that the GNPs exhibited strong absorption peaks at 531 nm resulting from their characteristic surface plasmon resonance, as shown by Figure 1D. 

### 2.2. Gold Nanoparticles Association with U87 GBM Cells

**Figure 2:**
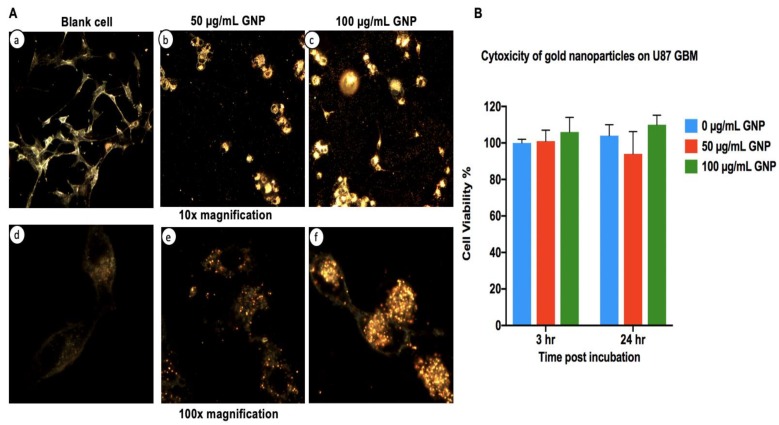
Gold nanoparticle association and cytotoxicity with U87 GBM cells. Figure 2**A** shows images from dark field microscopy under 10× magnification (panels **a**–**c**); a large number of gold nanoparticles were observed in association with the cells at the 100 μg/mL exposure level. Substantial amounts were also seen in 50 μg/mL samples. Under 100× magnification (panels **d**–**f**), increased GNP association with cells is observed with increasing concentrations of GNPs. Figure 2**B** shows an MTS viability assay of U87 GBM cells pretreated with increasing concentrations of GNPs at 3 and 24 h, respectively. No significant difference (as tested by ANOVA, see Methods) was observed between the three groups. Error bars represent the standard deviation of mean for three independent repeats (*n* = 3).

Exposure and biocompatibility of GBM cells to GNPs have previously been reported [19]. The goal of dark field microscopy in this study is to confirm the interaction of GNPs by the U87 GBM cells. Figure 2A confirms this with an observation of GNP association with cells evident at all tested concentrations (25, 50, and 100 μg/mL). Higher concentrations correlated with increased association, with 100 μg/mL GNPs having a substantially increased density of GNP clustering within the GBM cells. From these results, we were unable to definitively ascertain GNP uptake in cytosol compared to the nucleus. Notably, a nuclear association may be required for the optimal impact of Auger emission [20].

### 2.3. Cytotoxicity of Gold Nanoparticles in the Absence of Radiation

Before the impact of radiation on cells with GNPs can be ascertained, it is important to establish the “background” level of toxicity associated with exposure of the cells to the GNPs alone, as shown by Figure 2B. U87 GBM cells were incubated with increasing concentrations of GNPs and cell viability was assessed with MTS assays at 3 and 24 h, respectively. Cell viability remained greater than 90% in all GNP treated groups, 50 and 100 μg/mL GNP, with no statistically different viability levels evident in any group (at 3 h, *p* = 0.28; 24 h, *p* = 0.261) and no dose toxicity relationship evident. We also observed increased gold clustered grouping around cellular compartments and this appeared as cells “clumping” with increasing concentrations of GNPs at 10× magnification, as seen in Figure 2A. This can be further confirmed as distribution of the gold in the cell rather than cell aggregation as morphology can be seen to be unchanged in the 100× magnification images by examining the less bright (i.e., non-gold containing) regions of the cells at both 50 and 100 μg/mL.

### 2.4. Clonogenic Assay and Sensitization Enhancement Ratio

**Figure 3:**
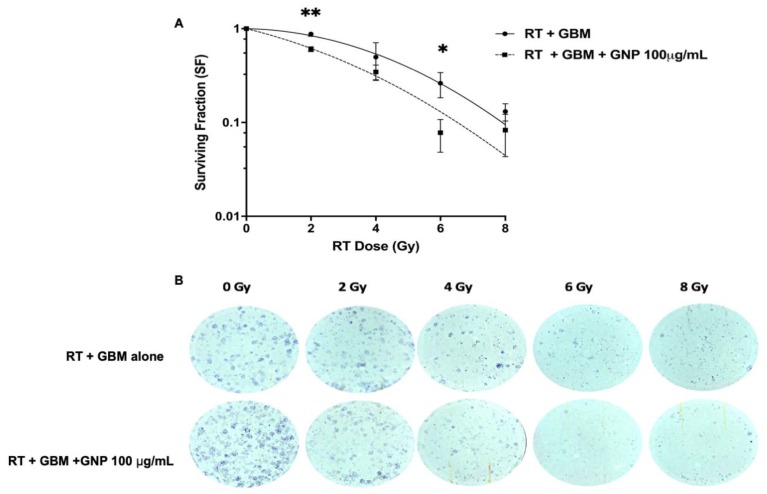
Radiosensitization of U87 GBM by gold nanoparticles. Figure 3**A** represents the survival curves for U87 GBM cells irradiated with 6 MV X-rays. Data were fitted based on a linear quadratic model comparing the non-GNP treated group and the GNP (100 μg/mL) treated group. Post irradiation, 2000 cells were counted and plated. Error bars represent the standard deviation of mean for three independent experiments. Significance tested by multiple *t*-test, * = *p* < 0.05 and ** = *p* < 0.01. Figure 3**B** shows representative images of the colonies for the GBM alone and GBM + GNP 100μg/mL groups at radiation dose 2, 4, 6, and 8 Gy. In the GBM alone group, the number of colonies formed at day 10 were 74, 66, 54, 19, and 10 at 0, 2, 4, 6, and 8 Gy, respectively. In GBM + GNP 100 μg/mL wells, the number of colonies formed were 92, 62, 42, 8, and 8 at the respective radiation dose points.

The most robust means of quantifying radiation-induced cell death is to study the ability of cells to form and grow colonies post-exposure (see Methods). U87 GBM cells incubated with GNPs (100 μg/mL) demonstrated a radiosensitization effect compared to those exposed to RT alone, as shown by Figure 3A and the images in Figure 3B. Notably, the survival fraction (SF) at 2 Gy was significantly higher for the GBM alone group (mean (M) = 0.88, SD = 0.05) versus the GNP (100 μg/mL) treated group (M = 0.61, SD = 0.06, *p* = 0.004). The sensitization enhancement ratio with GNP at 2 Gy (SER_2Gy_) was therefore 1.45. At 6 Gy, substantial and significant enhanced cell kill was seen with the GNP group giving a SF value 4-fold lower (M = 0.07, SD = 0.05, *p* = 0.03) compared to GBM alone (M= 0.26, SD = 0.008). Interestingly, this level of kill was not matched even by exposure to 8 Gy in the GBM alone group. At 4 Gy, the trend of decreased SF was also seen in the GNP group compared to GBM, but the effect did not reach significance (GNP group M = 0.35, SD = 0.11 vs. GBM alone group M = 0.49, SD = 0.21, *p* = 0.33), due to the large variation at this dose. Finally, at 8 Gy, the dose response was noted again, showing a similar trend with increased cell kill in the GNP group (M = 0.083, SD = 0.068, *p* = 0.322) compared to GBM alone (M = 0.131, SD = 0.027) but not to a statistically significant level.

## 3. Discussion

In this study, we have demonstrated a significant radiosensitization effect with SER_2Gy_ 1.45 (*p* = 0.004) with 42 nm GNPs at high concentrations (100 μg/mL) in the U87 GBM cell line with megavoltage radiation. A simple and fast one-step synthesis method was used to fabricate the GNPs without the need for complex surface modifications to achieve the radiosensitization effect. Dark field microscopy revealed high co-localization and biocompatibility of the GNPs within the GBM cells. The rationale behind utilizing 42 nm GNP in this experiment was based on a study by Chitrani’s group that demonstrated 50 nm GNPs exhibited the highest radiosensitization in HeLa cells compared to 14 and 74 nm GNPs when irradiated with 220 kVp X-rays; DEF of 1.43 compared to 1.2 and 1.25, respectively [21]. In addition, various reports have shown the relative safety of GNPs in the range of 20–50 nm in different cell lines [22,23]. A recent in vitro study demonstrated no GNP-associated toxicity on HepG2 cells when incubated with 20 and 50 nm GNPs [24]. However, in the same study, 5 nm GNPs exerted significant genotoxicity in a dose-dependent manner [24]. Longitudinal cell viability studies have also confirmed that GNPs are biocompatible at 48 hours post-incubation in various cancer cell lines [25,26].

From our literature review, we did not find any in-vitro study investigating the MV radiosensitization in U87 glioblastoma cell lines. We feel that this is an important area of investigation in view of the radio-resistance that is commonly seen in GBM, compared to other cancer types, and the fact that MV radiation is more clinically appropriate than kV. The reason for this is based on the fact that GBMs are deep-seated tumors with extensive resection margins and the percentage dose depth penetration with kV energies fall off significantly within 10 cm, with less than 20% of the prescribed dose being deposited at the depth less than 10 cm [6].

Several groups have reported significant MV radiosensitization in various other cancer cell lines. Jain and co-workers were one of the first groups to demonstrate significant radiosensitization in MDA-MB-231 cells; SER_2Gy_ 1.29 and 1.16 with 6 MV and 15 MV X-rays, respectively [15]. In-vitro studies by Chithrani also demonstrated similar outcomes, i.e., radiosensitivity enhancement factor (REF) 1.17, with 50 nm GNPs incubated in HeLa cells using 6 MV X-rays [21]. Similarly, Liu et al. demonstrated significant radiosensitization with 6.1 nm PEGylated GNP incubated in CT26 cells and irradiated with 6 MV X-rays [13]. Based on the survival curves in the paper, we deduced the estimated SER_2Gy_ (ratio of survival fractions without and with GNPs) was 1.40 for 500 μM PEGylated GNP, which was similar to the SER_2Gy_ value of 1.45 that we observed in this study [13]. Interestingly, Liu also demonstrated a direct correlation with the radiosensitization effect and increasing GNP colloidal concentration [27].

**Figure 4:**
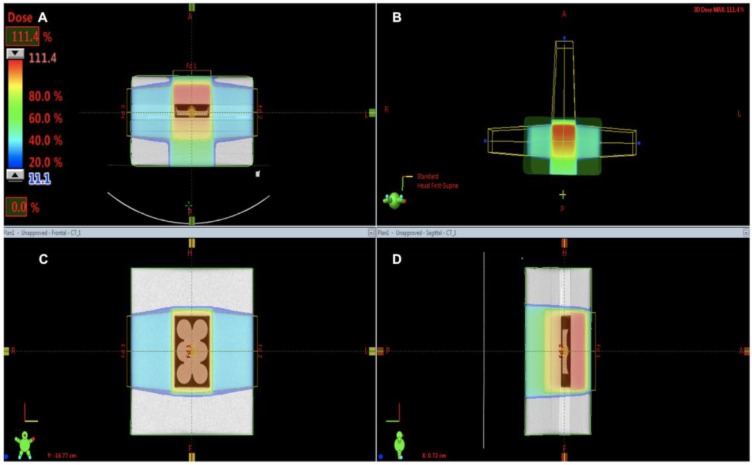
Irradiation setup. Demonstrates the dosimetry on the Eclipse™ treatment planning system using a computed tomography (CT) simulation. (**A**) shows a sagittal view of the CT simulation of well plates. The well plates are within the 95% isodose line (orange), indicating that at least 95% of the prescribed dose is homogenously delivered to the GBM cells. (**B**) shows the 3-field technique (2 lateral opposed fields and a single anterior field). (**C**) is an axial view to demonstrate the field size encompasses all 4-well plates. (**D**) shows the lateral view of bolus and plastic phantom.

These results are aligned with our findings that, indeed, MV radiation does exert significant dose enhancement at the target site. However, we also noted contrasting reports regarding MV radiosensitization with GNPs. Sara et al. reported results of a direct comparison of radiosensitization achieved between 160 kV versus 6 MV X-rays on platinum-sensitized F98 glioma cells [28]. She demonstrated DEF 1.81 following irradiation with 160 kV X-rays compared with 1.14 for 6 MV photons [28]. In this scenario, we would expect higher radiosensitization with MV X-rays, especially when delivered concurrently with a platinum radiosensitizer. Several reasons could explain these findings. Firstly, from our experience, radiotherapy dosimetry for MV planning using a single anterior or posterior field generally results in significant dose heterogeneity; hence, these results may not be reflective of a homogenously irradiated sample [28]. Secondly, RT was delivered using a single dose of 7 Gy which may indeed be a maximum tumoricidal dose and GNP may not add any meaningful enhancement at such a high dose [28]. We also noted similar findings with our colony-forming assay at the 8 Gy dose point, as shown in Figure 4. 

Despite these findings, it is important to note that there is a lack of a unified metric to characterize radiation effects in the literature. This has led to vast diversity in defining radiosensitization. Studies have used the dose enhancement factor (DEF), the radiosensitivity enhancement factor (REF), and the dose modifying factor (DMF) interchangeably to describe radiosensitization effects as a ratio of doses to achieve the same effect, hence making it difficult for direct comparison [6,11,15,29]. Our rationale for using SER_2G_ is to reflect the clinical setting, as the standard radiotherapy regimen for glioblastoma is to deliver 60 Gy in 30 fractions (2 Gy per fraction) to the target volume [2].

The strength of this study is as follows: we used a simple yet robust model to study radiosensitization on bare GNPs using a clinical LINAC. We strongly advocate the use of a simple 3-field RT delivery technique as it overcomes any dose inhomogeneity that would otherwise result from air gaps between the lid and the medium, as shown by Figure 4, and so would help standardize experiments performed at different centers using a LINAC to irradiate samples. Secondly, we used the “gold standard” clonogenic assay method to determine primary cell survival curves with good accuracy using 4 RT dose points (2–8 Gy). The clonogenics also validated our 3-field model as we observed a tumor dose response, as shown by Figure 3B.

A number of limitations need to be mentioned. Firstly, although a demonstration of co-localization of GNPs with GBM cells was achieved, it is difficult to assess the true yield of intracellular uptake based on dark field experiments and there is no confirmation that the GNPs are located in the perinuclear space where they generate the greatest impact [6]. Hence, this issue may be addressed in future studies to optimize the effect achieved, potentially by surface modification of the gold. Secondly, there was a lack of comparison to the GNP groups with different concentrations so it would be difficult to establish radiosensitization correlation to GNP concentration. Finally, functional assays, such as H2AX, are needed as it would provide more insights into the mechanism cell kill. However, due to the complex nature of metal oxide nanoparticles and their interactions with photons, we wanted to first establish a robust model prior to delving more into understanding the mechanism of action. It is also important to comment on the limitations of monolayer cell cultures which may not reflect the true radioresistant subpopulation of “GBM stem-like cells” (GSC) that reside in the hypoxic core of the high dose RT treatment field [30]. Hence, future in vitro studies should be conducted using 3D cell culture as this captures the true intratumor heterogeneity and microenvironment of GBM [30]. In addition, direct comparisons using different GBM cell lines and possibly different cancer cell lines would provide for interesting correlations in terms of intrinsic radiosensitivity to GNPs. Controls with fibroblasts should also be included to further evaluate toxicities in healthy tissue.

In conclusion, we have demonstrated a significant GNP radiosensitization in U87 GBM cell lines using a clinical platform. In addition, we demonstrated the advantage of using a 3-field RT technique that has not been described prior to this study using in-vitro models. Although these results are promising, further understanding of the mechanism of radiosensitization is needed. For example, it would be interesting to quantify ROS generation and DNA double-strand breaks using a robust experimental model, similar to this experiment. Finally, additional in vivo experiments will hopefully help define the ultimate clinical utility of delivering GNPs to tumor sites.

## 4. Materials and Methods

### 4.1. Cell Lines and Culture

A U87 human glioblastoma (GBM) cell line was obtained from American Type Culture Collection (ATCC, VA, USA), cultured using 20 mL of Dulbecco’s modified Eagle’s medium (DMEM), supplemented by 10% fetal bovine serum, 1% antibiotic mixture containing penicillin (Sigma-Aldrich, St. Louis, MO, USA), and streptomycin (Sigma-Aldrich). The cells were plated in a T75 flask (Thermo Fisher, St. Louis, MO, USA) and allowed to form a primary monolayer and stored at a humidified atmosphere of 37 °C with 5% CO_2_. The medium was changed every two days. Once the cells reached 80% confluency, the medium was removed and the cells were washed three times with 10 mL of Phosphate-buffered saline (PBS). Subsequently, the cells were detached from the flask using 4 mL of trypsin-EDTA and stored in a 37 °C incubator with a humidified atmosphere of 5% CO_2_ for 5 min. Next, the cells are suspended in 6 mL of DMEM and split (1:10), transferring 1 mL of the suspension to a new T75 flask with 20 mL of DMEM.

### 4.2. Gold Nanoparticle Synthesis and Characterization

Gold nanoparticles (GNPs) were fabricated following the classical method introduced by Turkevich [31]. One hundred mL 0.01% chloroauric acid (HAuCl_4_:4H_2_O) solution was refluxed and 1 mL 1% sodium citrate solution was added, respectively, to the boiling solution. The reduction of gold ions by the citrate ions was completed after 5 min. The solution was further boiled for 30 min and then left to cool at room temperature. Impurities were removed using dialysis with a membrane of 12–14 kD molecular weight cut-off (MWCO) for 72 h against ultrapure water. This step ensured the removal of all the ions and other unreacted reactants in the colloidal solution.

The hydrodynamic diameter (HD) distributions and Zeta potential of gold nanoparticles were determined by dynamic light scattering (DLS) using a NanoZS Zetasizer (Malvern, UK). The DLS data were acquired in the phase analysis light scattering mode at 25 °C, and the sample solutions were prepared by dissolving the GNPs in 10 mM phosphate-buffered saline (PBS) solution (pH = 7.0). The size and morphology of GNPs were analyzed by transmission electron microscopy (TEM) using a Hitachi HF-2000 field emission high-resolution TEM operating at 200 kV. The stability of GNPs was evaluated using optical UV–vis absorption spectra. It was recorded on a UV-1800 spectrophotometer (Shimadzu, Tokyo, Japan). The photoluminescence (PL) spectra were measured by an F4600 fluorescence spectrophotometer (Hitachi, Tokyo, Japan)

### 4.3. MTS Assay

To assess for GNP toxicity on GBM alone, cell viability was measured at 3 and 24 h post GNP exposure using a CellTiter 96 Aqueous One Solution cell proliferation assay kit MTS assay (Promega, Madison, WI, USA). Cells of 5 × 10^3^ (100 μL per well) were seeded in triplicates with 200 μL of DMEM in the 96-well plates and stored in the incubator overnight in a humidified atmosphere at 37 °C with 5% CO_2_. Next, the cells were washed with PBS and the medium was replaced with fresh medium containing different concentrations of GNPs (50, 100 μg/mL) and medium only (GBM cell with no GNP). After 3 h, the medium was removed and cells were washed with PBS twice. Next, 20 μL MTS was added to each well and incubated for 1 h. The optical density (OD) was recorded at 490 nm in a 96-well plate reader (Biorad, Watford, UK). The experiment was repeated to assess for cytotoxicity at 24 h. Cell viability was expressed as a percentage of the absorbance value of the GNP treated group to the GBM alone group. Data was a representation of 3 independent experiments (*n* = 3) for each group (3 and 24 h post GNP exposure).

### 4.4. Dark Field Microscopy

In accordance with the work of Manjari’s group, who demonstrated the uptake of GNPs into U87 GBM, the following procedure was followed to load cells with GNPs [19]. At 80% confluence, a total of 1 × 10^4^ cells were seeded on 6-well plate coverslips for 24 h. Next, they were dosed with different concentrations of GNP (50 and 100 μg/mL) and left overnight at 37 °C with 5% CO_2_. The following day, the cells were washed two times with PBS to remove excess GNPs and fixed with 4% formaldehyde for 5 min and washed again three times with PBS. Mounting solution (oregano limonene) was added and left overnight to dry. The absence of excess GNPs in media or on plastic-ware validated the effectiveness of the washing and the punctate distribution of the GNPs within the cells indicated cell uptake rather than the residence of the particles on the surface. Dark field images were taken using an Olympus BX-53 with a dry condenser (U-DCD, Essex, UK) and an oil immersion condenser (U-DCW, Essex, UK) with objective 10× and 100× lens (NA1.2–1.4), respectively.

### 4.5. Irradiation Setup

Radiation doses were delivered as a single fraction (2, 4, 6, and 8 Gy) using 6 MV X-rays with TrueBeam® Linac (Varian, Radiotherapy System, Palo Alto, CA, USA) at a dose rate of 600 MU/min. Source-axis-distance (SAD) = 100 cm, and 10 × 15 cm^2^ field size. A Bolus of 5 cm thickness was placed on the top of the plate to serve as a build-up material for the 6 MV beam. A plastic phantom 30 × 30 cm^2^ with 8 cm thickness was placed below for the back scatter dose. The setup was put through a computed tomography (CT) simulation. Radiotherapy planning (Eclipse™ treatment planning system, Palo Alto, CA, USA) was done using a 3-field planning technique (2 lateral opposed fields and a single anterior field) to provide a homogenous dose distribution. Well plates were encompassed by the 95% isodose line. The setup is shown in Figure 4. 

### 4.6. Clonogenic Assay

Clonogenic assay was performed to compare the two groups: GBM cells treated with GNPs (100 μg/mL) versus GBM cells alone. Experiments were carried on a 6-well plate. At 80% confluence, 3 × 10^4^ cells were seeded per well for 24 h. Next, they were incubated with the GNPs (100 μg/mL) overnight. Subsequently, they were irradiated the following day. Radiation was delivered using single doses: 2, 4, 6, and 8 Gy using a LINAC. Immediately after irradiation, 2000 cells were seeded in each well plate and incubated at 37 °C with 5% CO_2_ for 10 days or until colonies of greater than 50 cells had been formed. After sufficient colonies had formed, DMEM was removed, washing performed three times with 1 mL PBS, and 500 µL of 10% (*v/v*) neutral-buffered formalin with 50 µL of crystal violet added to each well for 60 min at room temperature. Next, the formalin–crystal violet mixture was removed with repeated washing using deionized water. Colonies of 50 cells or more were counted manually and surviving fractions were calculated, dividing the plating efficiency of GNP (100 μg/mL) treated cells by plating efficiency of GBM alone (No GNP). The experiment was repeated for an additional two times to have a total of three independent experiments (*n* = 3). Survival fraction (SF) was calculated based on the following equations [32]:

(1) $$PE=\frac{Number\;of\;colonies\;counted\;}{Number\;of\;cells}\;\times \;100$$

where PE is the plate efficiency. All the PEs of the treated samples were normalized to that of the control non-irradiated plates.

(2) $$SF=\frac{PE\;of\;treated\;sample}{PE\;of\;control\;}\;\times \;100$$

Survival fraction (SF) results were fit with a linear-quadratic (LQ) model, represented by Equation (2). Data was generated using GraphPad Prism 8.0 and plotted on a log (% survival) vs. dose plot.

(3) $$S=e^{-\alpha D-\beta D{}^{2}}.$$

where S is the survival fraction, α and β are the model constants, and D is the absorbed dose in Gy. 

### 4.7. Sensitization Enhancement Ratio

The sensitization enhancement ratio (SER_2GY_) was determined as the ratio of survival fractions without and with GNPs (100 μg/mL) at 2 Gy. This was based on previous data that have shown that the initial slope of the survival curve, rather than the final slope, correlates well with clinical outcomes [33,34]. 2 Gy also represents the typical individual dose of conventional radiotherapy fractionation delivery [35].

### 4.8. Statistical Analysis

Differences between GNP-treated and control groups were calculated using an independent sample *t*-test and one-way analysis of variance (ANOVA) with a post-hoc Tukey test using SPSS, with a *p*-value of less than 0.05 set to be considered significant. The correlation *r*^2^ values were calculated from the Pearson correlation coefficient using Prism 8.0 (GraphPad Software, San Jose, CA, USA). For the clonogenic assay, data were the representation of 3 independent experiments (*n* = 3). All results were expressed as mean ± standard deviation (SD).
